# Dietary Sutherlandia and Elderberry Mitigate Cerebral Ischemia-Induced Neuronal Damage and Attenuate p47phox and Phospho-ERK1/2 Expression in Microglial Cells

**DOI:** 10.1177/1759091414554946

**Published:** 2014-10-09

**Authors:** Dennis Y. Chuang, Jiankun Cui, Agnes Simonyi, Victoria A. Engel, Shanyan Chen, Kevin L. Fritsche, Andrew L. Thomas, Wendy L. Applequist, William R. Folk, Dennis B. Lubahn, Albert Y. Sun, Grace Y. Sun, Zezong Gu

**Affiliations:** 1 Interdisciplinary Neuroscience Program, University of Missouri, Columbia, MO, USA; 2 Center for Translational Neuroscience, School of Medicine, University of Missouri, Columbia, MO, USA; 3 Center for Botanical Interaction Studies, University of Missouri, Columbia, MO, USA; 4 Department of Pathology and Anatomical Sciences, University of Missouri, Columbia, MO, USA; 5 Harry S. Truman Memorial Veterans’ Hospital, Columbia, MO, USA; 6 Department of Biochemistry, University of Missouri, Columbia, MO, USA; 7 Department of Animal Sciences, University of Missouri, Columbia, MO, USA; 8 Southwest Research Center, University of Missouri, Mt. Vernon, MO, USA; 9 Missouri Botanical Garden, St. Louis, MO, USA

**Keywords:** botanical diet, global cerebral ischemia, microglia, oxidative stress, p47phox, phospho-ERK1/2

## Abstract

Sutherlandia (*Sutherlandia frutescens*) and elderberry (*Sambucus* spp.) are used to promote health and for treatment of a number of ailments. Although studies with cultured cells have demonstrated antioxidative and anti-inflammatory properties of these botanicals, little is known about their ability to mitigate brain injury. In this study, C57BL/6 J male mice were fed AIN93G diets without or with Sutherlandia or American elderberry for 2 months prior to a 30-min global cerebral ischemia induced by occlusion of the bilateral common carotid arteries (BCCAs), followed by reperfusion for 3 days. Accelerating rotarod assessment at 24 h after BCCA occlusion showed amelioration of sensorimotor impairment in the mice fed the supplemented diets as compared with the ischemic mice fed the control diet. Quantitative digital pathology assessment of brain slides stained with cresyl violet at 3 days after ischemia/reperfusion (I/R) revealed significant reduction in neuronal cell death in both dietary groups. Immunohistochemical staining for ionized calcium-binding adapter molecule-1 demonstrated pronounced activation of microglia in the hippocampus and striatum in the ischemic brains 3 days after I/R, and microglial activation was significantly reduced in animals fed supplemented diets. Mitigation of microglial activation by the supplements was further supported by the decrease in expression of p47phox, a cytosolic subunit of NADPH oxidase, and phospho-ERK1/2, a mitogen-activated protein kinase known to mediate a number of cytoplasmic processes including oxidative stress and neuroinflammatory responses. These results demonstrate neuroprotective effect of Sutherlandia and American elderberry botanicals against oxidative and inflammatory responses to cerebral I/R.

## Introduction

Throughout human history, many natural products from plants have been suggested to promote human health and manage disease symptoms, and some have been developed into modern-day drugs. Studies in recent years have documented antioxidative and anti-inflammatory properties of fruits and vegetables and herbs and indicate that some of these can maintain brain health during aging (Galli et al., 2002; Sun et al., 2008). It is important to understand the molecular mechanisms underlying their mode of action.

Sutherlandia (*Sutherlandia frutescens* [L.] R. Brown or *Lessertia frutescens* [L.] Goldblatt & J.C. Manning), also known colloquially as cancer bush, is widely used in southern African traditional and contemporary remedies for a variety of chronic ailments, including cancer, arthritis, digestive disorders, and diabetes, and more recently, behavioral symptoms of HIV/AIDS such as depression and anxiety (Mills et al., 2005; van Wyk and Albrecht, 2008). Studies with cell and animal models have demonstrated its antioxidant and anti-inflammatory properties (Fernandes et al., 2004; Ojewole, 2004; Katerere and Eloff, 2005; Kundu et al., 2005; Faleschini et al., 2013; Jiang et al., 2014). Although some evidence supports Sutherlandia’s benefit for mitigating stress (Prevoo et al., 2004) as well as drug-induced seizures (Ojewole, 2008), little is known about its broader effects against neurodegenerative diseases and stroke. Results from a randomized, double-blind, placebo-controlled trial in healthy adults of consumption of Sutherlandia for 3 months showed it was well tolerated (Johnson et al., 2007).

Consumption of elderberry including the North American subspecies (*Sambucus nigra* L. subsp. *canadensis* [L.] Bolli) has increased in recent years, mainly for its claimed ability to combat symptoms of common flu and other viral infections (Zakay-Rones et al., 1995; “*Sambucus nigra* (elderberry),” 2005; Vlachojannis et al., 2010). Elderberries are widely cultivated in Europe, Asia, North Africa, and North America (“*Sambucus nigra* (elderberry),” 2005). Elderberry fruit contains flavonoids and anthocyanins (Lee and Finn, 2007; Thomas et al., 2013), which are reported to have beneficial effects of human health, especially cardiovascular functions and anticarcinogenic, antiviral, and anti-inflammatory effects (Prior and Wu, 2006; Zafra-Stone et al., 2007). Cyanidin-3-glucoside, one of the most common anthocyanins of berries, was shown to ameliorate ethanol-induced neurotoxicity in developing brains and protect against focal cerebral ischemia in mice (Ke et al., 2011; Min et al., 2011). There is further evidence suggesting the ability of berries to prevent age-associated oxidative stress and to improve neuronal and cognitive functions in animal models (Galli et al., 2002). Despite the increasing interest regarding these secondary metabolites, little is known whether elderberries alleviate stroke damage.

Stroke is the second leading cause of death worldwide and is the primary cause of acquired disability in the United States (Davis and Donnan, 2012). Although the pathophysiology of ischemic damage is complex, extensive studies have focused on the underlying mechanisms of oxidative stress and inflammatory responses following ischemia/reperfusion (I/R; Chen et al., 2011a, 2011b). Studies have demonstrated the role of NADPH oxidase and activation of the mitogen-activated protein kinase (MAPK) pathways in production of reactive oxygen species (ROS) and signaling events leading to mitochondrial dysfunction and activation of apoptotic pathways (Chen et al., 2011a; Yoshioka et al., 2011a). Among the various *in vivo* models of cerebral ischemia, the murine bilateral common carotid artery (BCCA) occlusion model has been documented to cause damage in the hippocampal and striatal neurons (Lin et al., 2000; Wang et al., 2005b; Yoshioka et al., 2011a). Previous studies with the gerbil global BCCA occlusion model demonstrated protective effects of botanicals such as curcumin and grape polyphenol extracts against neuronal cell death and glial cell activation in the hippocampal CA1 area (Wang et al., 2002; Simonyi et al., 2005; Wang et al., 2005b).

Both Sutherlandia and elderberry share the capacity to relieve oxidative stress and suppress inflammatory responses. In this study, the murine global cerebral ischemia model was used to demonstrate that dietary supplementation by Sutherlandia and American elderberry offer protection against ischemia-induced neuronal damage and glial cell activation and neurobehavioral dysfunctions.

## Materials and Methods

### Materials

Antibodies used for immunohistochemical staining include rabbit anti-p47phox antibody (sc-14015; Santa Cruz Biotechnology, Santa Cruz, CA) and mouse anti-phospho-ERK1/2 (phosphorylated extracellular signal-regulated kinases 1/2) monoclonal antibody (9102; Cell Signaling, Beverly, MA). Cell-type-specific antibodies include rat anti-CD11b (cluster of differentiation molecule 11b, 550274; BD Biosciences, San Jose, CA) and rabbit anti-Iba-1 (ionized calcium-binding adapter molecule-1) antibodies (019-19741; Wako BioProducts, Richmond, VA) for microglia, and rabbit anti-glial fibrillary acidic protein (GFAP) antibodies (G9269; Sigma-Aldrich, St. Louis, MO) for astrocytes. Secondary antibodies include goat anti-mouse IgG-Alexa488 (A11001), goat anti-rabbit IgG Alexa 488 (A110034), goat anti-mouse IgG Alexa fluor 594 (A11005), and goat anti-rat IgG Alexa fluor 594 (A11007; Life Technologies/Invitrogen, Carlsbad, CA).

### Animals, Diets, and Ischemia Protocol

Adult male C57Bl/6J mice (The Jackson Laboratory, Bar Harbor, ME) at age 8 weeks were housed 4/cage and maintained on a 12-h light/dark cycle (lights on at 7:00 a.m.) with unrestricted access to food and water. Prior to surgical BCCA occlusion, mice were fed for 2 months with a nutritionally complete experimental diet AIN93G with or without supplement of either 1% by weight of freeze-dried, ground Sutherlandia-dried vegetative material or 2% by weight of freeze-dried, ground whole ripe fruit of American elderberry, based on empirical estimates of mouse equivalents of human consumption (Wang et al., 2005c; Johnson et al., 2007; see Table 1 for complete composition of the control and test diets). Sutherlandia vegetative material was purchased from Big Tree Nutraceutical (Fish Hoek, South Africa) and stored at −20°C in an air-tight container in the dark. The elderberry fruits were harvested in 2010 from a germplasm repository in southwest Missouri (United States) and frozen in zippered plastic freezer bags. Berries were later de-stemmed and cleaned, lyophilized, and ground into fine powder before addition to diets. A mixture of several American elderberry genotypes was used in this study. Botanical vouchers confirming the taxonomic identity of plants were deposited into the herbaria of the University of Missouri or the Missouri Botanical Garden (St. Louis, MO). Average food intake was 2.6 ± 0.05 g/day/mouse; and average diet consumption was 0.106 ± 0.003 g/gram body weight/day. Weekly monitoring of body weight indicated no differences in weight between any of the groups at any time during the course of the study. Approved animal protocols were obtained, and all treatment steps were in accordance with University of Missouri and the National Institutes of Health guidelines for the Care and Use of Laboratory Animals.

**Table 1.** Composition of the Control AIN93G and Diets Supplemented With Sutherlandia and Elderberry.

Ingredient	Control diet		1% Sutherlandia diet		2% Elderberry diet	
	g/kg of diet	% of total	g/kg of diet	% of total	g/kg of diet	% of total
Cornstarch	397.3	39.73	387.3	38.73	387.3	37.73
Casein	200.0	20.00	200.0	20.00	200.0	20.00
Dextrose	132.0	13.20	132.0	13.20	132.0	13.20
Sucrose	100.0	20.00	100.0	10.00	100.0	10.00
Fiber (cellulose)	50.0	5.00	50.0	5.00	50.0	5.00
Mineral mix (AIN-93)	35.0	3.50	35.0	3.50	35.0	3.50
Vitamin mix (AIN-93G)	10.0	1.00	10.0	1.00	10.0	1.00
l-cystine	3.0	0.30	3.0	0.30	3.0	0.30
Choline bitartrate	2.5	0.25	2.5	0.25	2.5	0.25
Soybean oil	70.0	7.00	70.0	7.00	70.0	7.00
Food dye (color varies)	0.2	0.02	0.2	0.02	0.2	0.02
Elderberry (whole ripe fruit)^a^	–	–	–	–	20	2.00
Sutherlandia (dried leaves)^b^	–	–	10	1.00	–	–
Total	1,000	100	1,000	100	1,000	100

a York and Bob Gordon cultivars (60:40 ratio) were harvested at the peak of ripeness, freeze-dried, then ground into a fine powder, and mixed with an equal weight of corn starch to prevent clumping. The amount of this mix added to the diet was actually twice that shown in the table.

b Sutherlandia was sourced Big Tree Nutraceutical (21 First Avenue, Fish Hoek 7975, South Africa). Prior to its incorporation into the diet, the chopped leaves were grounded into a fine powder using a handheld coffee bean grinder.

For the study, mice were divided into four experimental groups: (a) sham animals with AIN93G control diet (Sham/CD, *n* = 7), (b) BCCA occlusion-induced ischemia with AIN93G diet (Isch/CD, *n* = 7), (c) BCCA occlusion-induced ischemia with AIN93G diet containing 1% Sutherlandia (Isch/SD, *n* = 7), and (d) BCCA occlusion-induced ischemia with AIN93G diet containing 2% elderberry (Isch/ED, *n* = 7). To conserve on animal numbers, sham operation was performed only on animals with the control diet. Animals were subjected to a transient global cerebral ischemia by BCCA occlusion as described previously with minor modifications (Lin et al., 2000; Chen et al., 2011a). To initiate the surgical protocol, mice were placed in a holding chamber and anesthetized with 4% isoflurane, and continuous anesthesia during surgery was maintained with 1% to 1.5% isoflurane in 70% nitrogen and 30% oxygen with a face mask. During the surgery, rectal temperature was monitored and maintained at 37 ± 0.5°C with a thermostat-controlled heating pad. BCCA occlusion was accomplished by applying microaneurysm clips on both common carotid arteries for 30 min followed by release of the clips and a 3-day reperfusion. Reestablishment of blood flow was confirmed by direct observation. Sham operation animals were subjected to the identical surgical procedures except for application of micro-aneurysm clips. After surgery, animals were placed in cages above a heating blanket to maintain rectal temperatures above 36°C for 1–2 h with active monitoring.

### Assessment of Sensorimotor Functions

**Figure 1.**
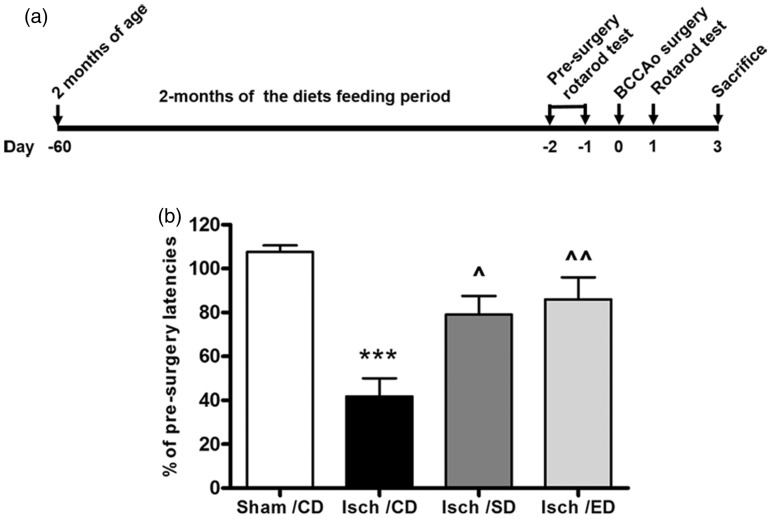
Supplementation of Sutherlandia and elderberry diets and assessment of motor coordination and balance functions in ischemic mice. (a) Experimental design for dietary feeding, rotarod assessment, and BCCA occlusion (BCCAo). (b) Accelerating rotarod assessment reveals amelioration of behavioral deficits from transient global cerebral ischemia by dietary supplement of Sutherlandia and elderberry. Rotarod performance is expressed as percent of time the mouse can stay on the accelerating rotarod compared with pre-ischemia as the baseline values. Four groups of mice were divided into (i) AIN93G control diet (Sham/CD), (ii) BCCA occlusion-induced ischemia with control diet (Isch/CD), (iii) BCCA occlusion-induced ischemia + Sutherlandia diet (Isch/SD), and (iv) BCCA occlusion-induced ischemia + elderberry diet (Isch/ED). Data are expressed as mean ± *SEM* (*n* = 7 for all groups). Statistical significance is denoted with ***p < .001 (compared with Sham/CD); *^p* < .05 and ^^*p* < .01 (compared with Isch/CD) by one-way ANOVA followed by Bonferroni’s posttest.

Assessment of sensorimotor functions was carried out by the rotarod test as described previously with modifications (Simonyi et al., 2012). Two days prior to BCCA occlusion, mice were trained on the rotarod (Med Associates, St. Albans, VT) in the acceleration paradigm (4–40 rpm/5 min) for three trials each day with a 30-min intertrial interval (see Figure 1(a) for the experimental design with BCCA occlusion set as Day 0). Latency is defined as the time spent on the accelerating rotating rotarod without falling off or gripping and spinning rather than walking. Preoperative baseline values were obtained by determining average of the three best performances. Postoperative testing (three trials) were performed 24 h after I/R, and the means were used for calculation of rotarod performance. Latencies measured at 24 h after I/R were expressed as % of performances reached before surgery.

### Brain Tissue Processing, Histochemical Staining, and Assessment of Neuronal Damage

Three days after I/R, mice were euthanized with isoflurane and brains were sectioned for histochemical staining and assessment of neuronal damage using a well-established protocol as described previously (Cui et al., 2012; Hadass et al., 2013). Briefly, mice were transcardially perfused with 4% paraformaldehyde in 100-mM phosphate buffer, and brains were dissected and preserved for 24 h in the same buffer. Serial coronal sections (40 µm) were obtained with a vibrotome (VT1200S, Leica Microsystems, Inc., Bannockbum, IL). In most instances, a total of 150 to 160 40-µm tissue sections from each brain were collected into 24-well plates. Serial brain sections 200-µm apart were mounted on poly-l-lysine-coated glass slides and followed by staining with cresyl violet, a stain for Nissl substance in the cytoplasm of neurons commonly used for assessment of neuronal cell death.

Pathological assessment of histological specimens was carried out in an unbiased manner using a high-throughput digital pathology system. Briefly, whole slide images (WSI) of the cresyl violet-stained brain sections were obtained using an automatic multifocus plane, high-throughput digital pathology system (Aperio ScanScope CS digital scanner, Vista, CA). Extent of neuronal damage in the brain sections were analyzed in a double-blind manner using the following criteria for the grading scale: 0: *no observable neuronal damage*; 1: *damaged neurons populate 0% to 25% of area*; 2: *damaged neurons populate 25% to 50% of area*; 3: *damaged neurons populate 50% to 75% of area*; and 4: *damaged neurons populate >75% of area* (Cui et al., 2012; Hadass et al., 2013).

### Fluorescence Immunohistochemistry

Fluorescence immunohistochemistry was carried out on brain sections for astrocytes (with GFAP) and microglia (with Iba-1 and CD11b; Hadass et al., 2013), as well as p47phox and phospho-ERK1/2. Briefly, fixed coronal sections from the area of interest were washed with phosphate-buffered saline (PBS) and permeabilized with 1% Triton X-100 in PBS for 30 min. Sections were incubated with Pro-Block (PBK125; ScyTek, Logan, UT) for 5 min to eliminate the need to match species with the fluorescence conjugated antibody, followed by 10% normal goat serum in 0.05% Triton X-100 in PBS for 60 min, and then overnight with 0.5% normal goat serum in 0.05% Triton X-100 in PBS containing the primary antibodies (GFAP, 1:500; CD11b, 1:400; Iba-1, 1:1000; p47phox, 1:400; and phospho-ERK 1:500). The next day, sections were washed and incubated in 0.05% Triton X-100 in PBS containing the appropriate fluorophore-conjugated secondary antibodies (1:300; goat anti-mouse IgG-Alexa488, goat anti-mouse IgG-Alexa594, and goat anti-rabbit IgG-Alexa488, goat anti-rat IgG Alexa594; Life Technologies/Invitrogen, San Diego, CA) for 2 h and counterstained in a solution of Hoechst dye 33342 (1:1000; H-3570, Life Technologies/Invitrogen, San Diego, CA). Fluorescence photomicrographs of the areas of interest were captured by a Leica DMI 6000B automated epifluorescence microscope (Leica Microsystems Inc., Buffalo Grove, IL), and the high magnification photomicrographs were processed using the AF6000 stitching program and intensity analysis with the ImageJ program. For quantification of immunofluorescence intensity, five representative areas in the striatum (coordinated approximately to Bregma 0.78 mm) were selected bilaterally (as indicated in the Figure 4(a)), and the microphotographic images of the immunostained brain sections were captured. All microphotographic images for examining each set of the experimental groups were taken under the same camera and microscope settings including the dimension, voxel size, and exposure parameters (intensity, exposure time and gain for each channel, as well as threshold values for black as 0, white as 255, and binning 1 × 1), except pERK which has threshold values from 99 to 255.

### Statistical Analysis

Data are presented as mean ± standard error of the mean (*SEM*). Results were analyzed by one-way analysis of variance (ANOVA) with Bonferroni’s posttest (V4.00; GraphPad Prism Software Inc., San Diego, CA). Statistical significance was considered for *p* < .05.

## Results

### Dietary Supplementation With Sutherlandia or Elderberry Ameliorated Motor Impairment in Mice After Transient Global Cerebral Ischemia

A time line of the experimental protocol, showing feeding of mice at 2 months of age, presurgery and postsurgery rotarod tests, and sacrifice for brain pathology is shown in Figure 1(a). In this experiment, sensorimotor functions in the sham, ischemia, and ischemia with Sutherlandia and elderberry diet groups were assessed using the accelerating rotarod paradigm. In the pre-ischemia rotarod tests, no differences were observed in sensorimotor functions between controls and the different dietary groups (Sham/CD, 254.0 + 5.9 s; Isch/CD, 245.6 + 10.7 s; Isch/SD, 247.0 + 12.5 s; Isch/ED, 275.7 + 6.2 s; *p* > .05, one-way ANOVA). Rotarod performance was unchanged in the sham-operated group but was significantly impaired in the ischemia group assessed 24 h after reperfusion (Figure 1(b)). Consumption of the Sutherlandia or elderberry supplemented diets for 2 months ameliorated the sensorimotor deficits by prolonging rotarod latencies.

### Consumption of Sutherlandia and Elderberry Diets Decreased Neuronal Damage After I/R

**Figure 2.**
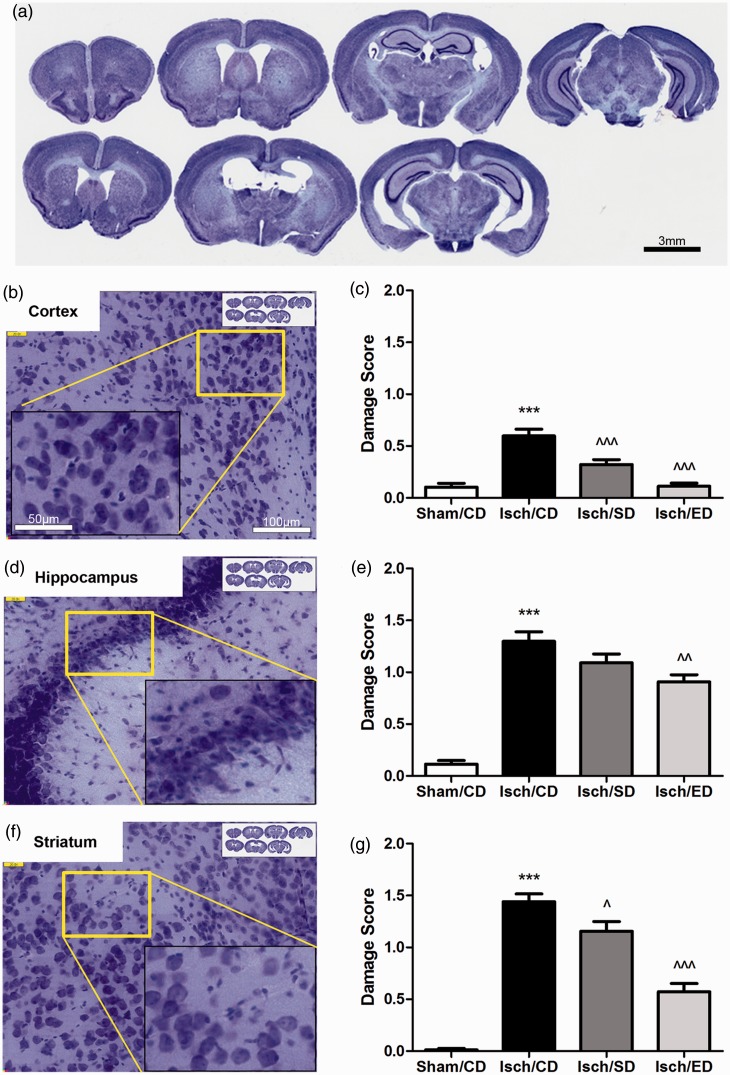
Consumption of Sutherlandia and elderberry diets ameliorates neuronal damage after transient global cerebral ischemia. (a) Representative photomicrographs of serial brain sections with each 960-µm apart; Scale bar = 3 mm. (b, d, and f) Representative images of neurons from cortex, hippocampus, and the striatal/thalamus/basal ganglia areas; Scale bar = 100 µm, and 50 µm for the inset. (c, e, and g) Graphical presentation of neuropathology scores in the respective brain regions (0 = *no damage*; 4 = *maximal damage*). Data are expressed as mean ± *SEM*, where *n* = 7 animals from each group. Statistical significance is denoted with ***p < .001 (compared with Sham/CD); *^p* < .05, ^^*p* < .01, and *^^^p* < .001 (compared with Isch/CD) with one-way ANOVA followed by Bonferroni’s posttest.

Cresyl violet staining of brain sections revealed substantial damage in neuronal morphology after BCCA occlusion (Figure 2(a)): While normal healthy neurons were round with pale-stained cytoplasm, many neurons in the ischemic regions in cortex, hippocampus, and striatum were angular in shape with condensed cell bodies (see higher magnification insets in Figures 2(b), (d), and (f), respectively). The zoomable WSI photomicrographs were acquired from approximately 30 to 35 serial tissue sections with 200-µm interval per brain. Damage scoring (0–4, based on the criteria described in the Materials and Methods section) for cerebral cortex, hippocampus, and striatum including thalamus and basal ganglia areas were carried out in a double-blind manner using the web-based ImageScope program. Quantitation of the cellular damage in each region revealed that I/R resulted in severe neuronal damage in the hippocampal but those in the striatum/thalamus/basal ganglia area and the cortical regions were also damaged albeit to a lesser extent (Figure 2(b) and (c)). Dietary supplementation with elderberry significantly decreased the neuronal damage in all the brain regions examined (Figure 2(c), (e), and (g)). To a lesser extent, decrease in neuronal damage was also observed in these brain regions on supplementation with Sutherlandia.

### Immunostaining of Microglial Cells and Astrocytes

**Figure 3.**
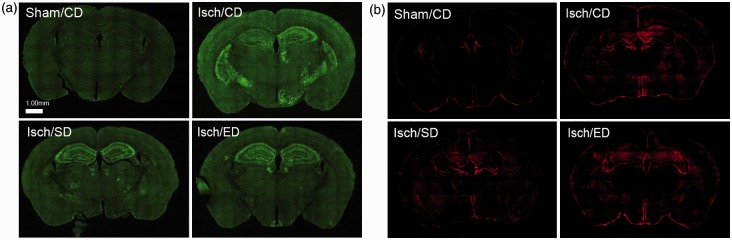
Brain sections immunostained with Iba-1 and GFAP. Microphotographs were generated by fluorescent microscope at resolution of 40× magnification for entire section regions, followed by automatic stitching of borders. (a) Representative brain fluorescent microscopic images of Iba-1 expression in the sham-operated mice with the control diet (Sham/CD), and the ischemic animals with the control diet (Isch/CD), Sutherlandia diet (Isch/SD), and elderberry diet (Isch/ED). (b) Representative whole-brain fluorescent microscopic images of GFAP expression in the sham-operated mice with the control diet (Sham/CD), and the ischemic animals with the control diet (Isch/CD), Sutherlandia diet (Isch/SD), and elderberry diet (Isch/ED). Scale bar = 1.00 mm in (a) and (b).

Neuronal damage after I/R is often accompanied with increased neuroinflammatory responses including astrogliosis and microglial activation (Wang et al., 2006; Brennan et al., 2009; Chen et al., 2011b). We assessed these responses 3 days after I/R by immunostaining brain sections with Iba-1 for microglia and GFAP for astrocytes. Immunofluorescent analysis with Iba-1 revealed low immunoreactivity in sham controls but a robust and widespread increase in the ischemic brain, especially in the hippocampal and striatal regions (Figure 3(a), Isch/CD). Both Sutherlandia and elderberry diets attenuated the Iba-1 immunoreactivity in the cortex and striatum (Figure 3(a), Isch/SD and Isch/ED).

We also examined brain sections immunostained with GFAP as a marker for activated astrocytes. Immunoreactivity of GFAP was low in sham controls but increased after I/R. The pattern for GFAP immunoreactivity appeared to be more spread out and diffuse as compared with those stained with Iba-1 immunoreactivity (Figure 3(b)).

### Sutherlandia and Elderberry Consumption Attenuated I/R-Induced Activation of Microglia but Not Astrocytes in the Striatum

**Figure 4.**
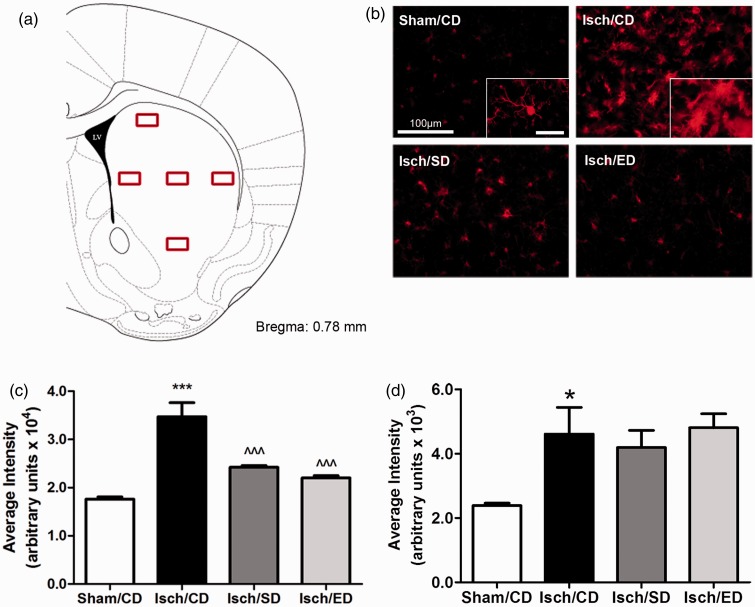
Consumption of Sutherlandia and elderberry diets attenuates activation of microglial cells but not astrocytes in the striatal regions after transient global cerebral ischemia. (a) Graphical illustration of the five representative areas selected bilaterally for captured intensity analysis. (b) Representative fluorescent microscopic images of Iba-1 immunoreactivity among all groups; Scale bar = 100 µm. Inset in Sham/CD panel shows representative cells of the ramified resting microglia, while inset in Isch/CD panel shows the amoeboid form of activated microglia; Scale bar = 25 µm. Quantitation of the average fluorescent intensity for Iba1 (c) and GFAP (d) immunoreactivity from five areas in the striatum (*n* = 5 for all groups). Data are expressed as means ± *SEM*. Statistical significance is denoted with *p < .05, ***p < .001 (compared with Sham/CD); and ^^^*p* < .001 (compared with Isch/CD) by one-way ANOVA followed by Bonferroni’s posttest.

Because the striatal region is more homogeneous, we selected five representative subregions for further examine cell immunoreactivity and morphology (Figure 4(a)). Immunoreactivity of Iba-1 expressing microglial cells in the sham-operated group appeared mostly in the resting ramified form with small round cell bodies and thin processes (Figure 4(b)), whereas those in the ischemic regions became amoeboid shape with irregular cell bodies and thick processes (see insets in Figure 4(b)). By assessing average fluorescence intensity of Iba-1 in these striatal regions, significantly lower immunoreactivity of microglia in mice fed the Sutherlandia or elderberry diets as compared with the ischemia group on the control diet (Figure 4(c)).

Similarly, fluorescence intensity of GFAP in the striatal regions indicated a significant increase in GFAP immunoreactivity after BCCA occlusion as compared with sham controls (Figure 4(d)). However, both Sutherlandia and elderberry diets did not attenuate GFAP immunoreactivity as compared with the ischemia group on the control diet (Figure 6(d)).

### Sutherlandia and Elderberry Consumption Inhibited I/R-Induced Increases in p47Phox Expression in the Striatum

**Figure 5.**
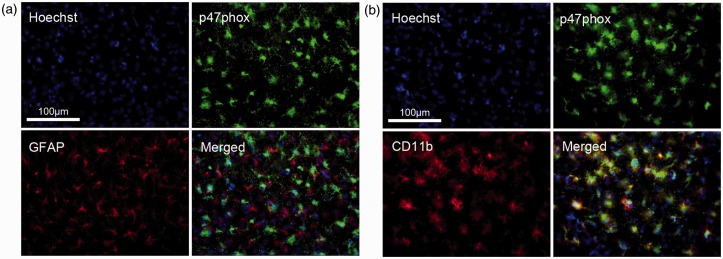
P47phox expression colocalized with microglia, and not astrocytes, in the striatum at 72 h after ischemia/reperfusion. (a) Fluorescent microscopic image of p47phox (green) and GFAP (red) staining showing no colocalization between the p47phox and astrocytes. (b) Fluorescent microscopic image of p47phox (green) and CD11b (red) staining showing colocalization between the p47phox and microglia; Scale bar = 100 µm in a and b.

A number of studies have demonstrated involvement of NADPH oxidase in ROS production during I/R (Wang et al., 2006; Chen et al., 2009). Double immunostaining of p47phox, an NADPH oxidase subunit, with GFAP for astrocytes and CD11b for microglial cells showed that p47phox immunoreactivity did not colocalize with the GFAP expressing astrocytes (Figure 5(a)), but instead with the CD11b expressing microglia (Figure 5(b)).

**Figure 6.**
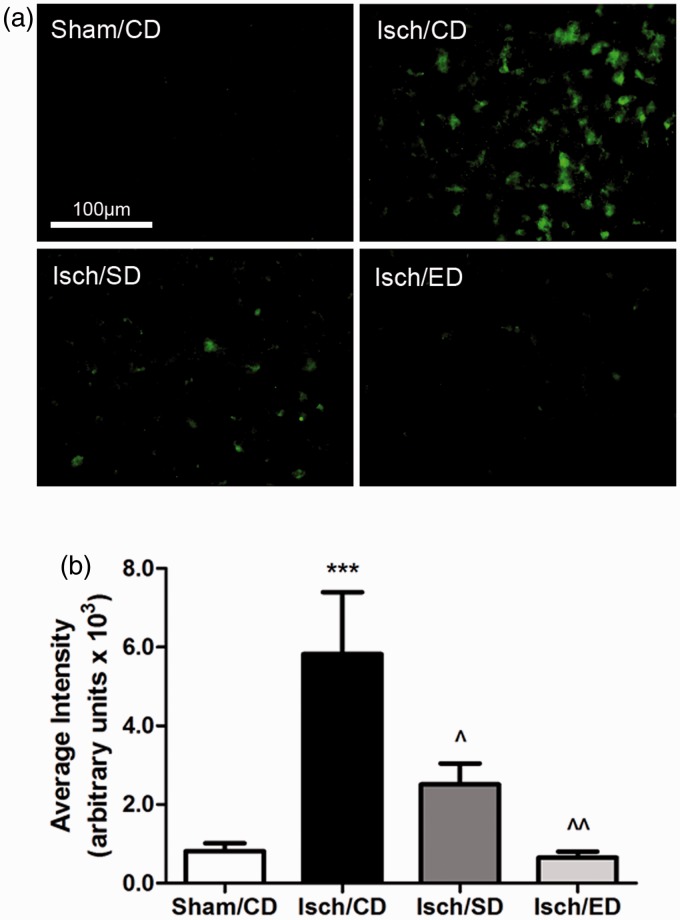
Immunostaining showing increase in p47phox expression in ischemic brain and attenuation by consumption of Sutherlandia and elderberry diets. (a) Representative fluorescent microscopic images of p47phox expression among four groups: Sham/CD, Isch/CD, Isch/SD, and Isch/ED; Scale bar = 100 µm. (b) Graphical presentation of the average fluorescent intensity from five areas of interest (*n* = 5 for all groups). Data are expressed as mean ± *SEM*. Statistical significance is denoted with ***p < .001 (compared with Sham/CD); *^p* < .05 and ^^*p* < .01 (compared with Isch/CD) by one-way ANOVA followed by Bonferroni’s posttest.

Quantitation of the p47phox immunoreactivity was carried out in the five selected areas in the bilateral striatal regions. A significant increase in p47phox immunoreactivity was observed in the striatum (Figure 6(a)) as well as in the hippocampus (data not shown) of the ischemic brain at 3 days after I/R. Measurement of fluorescent intensity from different areas in the striatal and caudate putamen region indicated that mice given either botanical diet had a significant decrease in p47phox immunoreactivity as compared with the ischemic group (Figure 6(b)).

### Sutherlandia and Elderberry Consumption Inhibited I/R-Induced Phospho-ERK1/2 Expression in Microglial Cells

**Figure 7.**
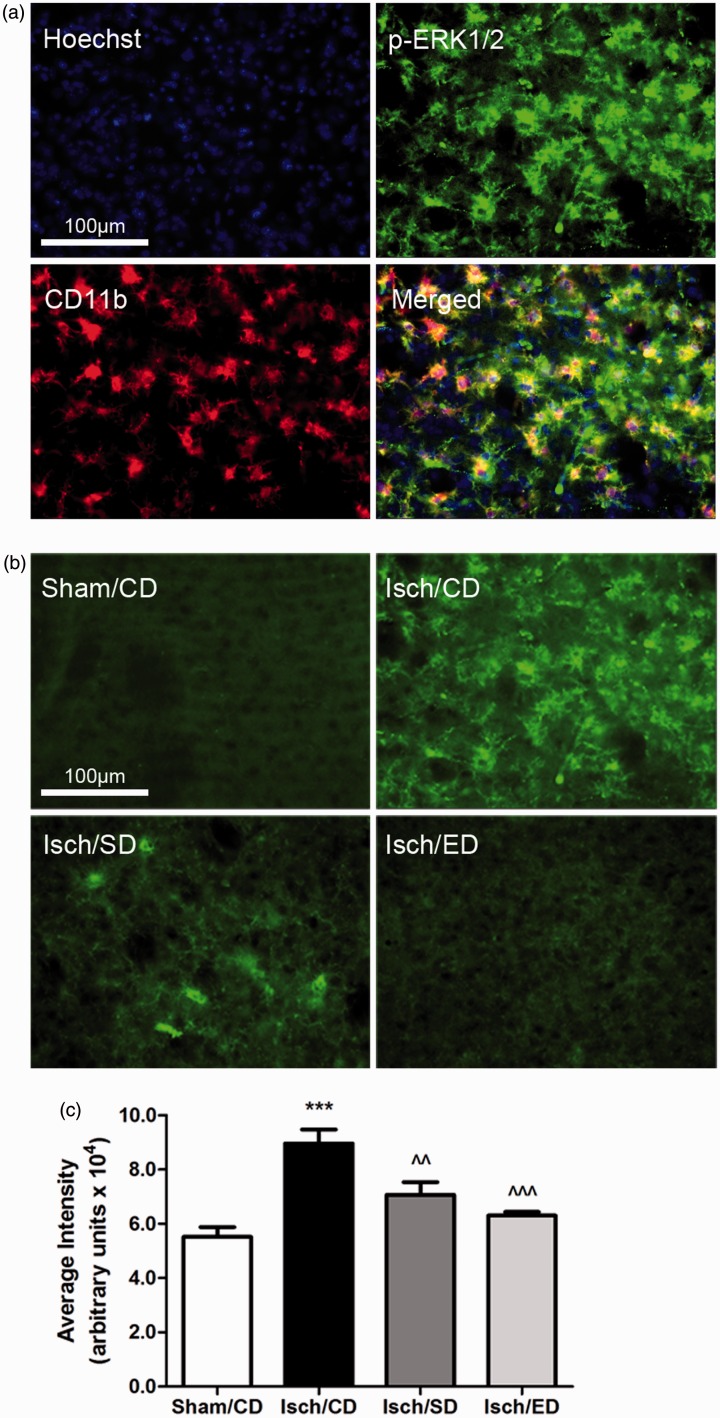
Increase in phospho-ERK1/2 expression in ischemic brain and attenuation by consumption of Sutherlandia and elderberry diets. The increased phospho-ERK1/2 (p-ERK1/2) expression is colocalized with microglia. (a) Fluorescent microscopic image of p-ERK1/2 (green) and CD11b (red) staining showing colocalization between the p-ERK1/2 and microglia in the Isch/CD condition. (b) Representative fluorescent microscopic images of p-ERK1/2 expression among all groups. Scale bar = 100 µm in (a) and (b). (c) Graphical presentation of the average fluorescent intensity from five areas of interest (*n* = 5 animals were selected randomly for seven in all groups). Data are expressed as mean ± *SEM*. Statistical significance is denoted with ***p < .001 (compared with Sham/CD); *^^p* < .01 and ^^^*p* < .001 (compared with Isch/CD) with by one-way ANOVA followed by Bonferroni’s posttest.

Recent studies with cultured microglial cells demonstrated involvement of ERK1/2 in the oxidative/nitrosative pathways associated with stimulation by lipopolysaccharide (LPS) and interferon gamma (IFNγ; Chuang et al., 2013). Based on the observations of increased p47phox immunoreactivity and activated microglial cells in striatum after I/R, we further examined phospho-ERK1/2 expression in the ischemic brain sections and compared immunoreactivity with the groups supplemented with Sutherlandia and elderberry diets. Double immunostaining for phospho-ERK1/2 and microglial marker CD11b indicated increased phospho-ERK1/2 immunoreactivity colocalized with many microglial cells in the striatum at 3 days after I/R (Figure 7(a)). Fluorescence intensity analysis showed significant decrease in phospho-ERK1/2 immunoreactivity in both dietary groups as compared with the ischemia control group (Figure 7(b) and (c)).

## Discussion

This study demonstrates that dietary Sutherlandia and elderberry mitigate behavioral deficits and pathology induced by BCCA occlusion in mice. These results agree with and extend our earlier studies using the Mongolian gerbil model demonstrating botanicals such as curcumin, apocynin, and grape polyphenols protect against ischemic damage (Wang et al., 2005a, 2005b, 2006, 2009). With the gerbil model, BCCA occlusion for 5 min causes extensive neuron cell death and glial activation in the hippocampal CA1 area 4 days after I/R (Wang et al., 2002). More recent studies by others using the murine model, and BCCA occlusion for 22 min resulted in damage in the hippocampal area as well as the striatal area (Yoshioka et al., 2011b). In the present study, we adopted the moderately severe murine BCCA occlusion model to investigate effects of dietary Sutherlandia and elderberry on I/R injury. BCCA occlusion for 30 min and followed by reperfusion for 3 days resulted in severe neuronal damage in the hippocampus, and sporadic cell death in the cortex as well as in the striatal/thalamus/basal ganglion regions. Using digital pathology and the 5-point scoring system on cresyl violet-stained brain sections, the results demonstrated dietary Sutherlandia and elderberry significantly mitigate the I/R-induced neuronal damage in all three brain regions. A behavioral test using the accelerating rotarod paradigm to monitor sensorimotor deficits of individual animals further showed that dietary supplementation of Sutherlandia and elderberry significantly ameliorated the I/R-induced motor/coordination deficits.

Cerebral ischemia not only causes damage to neurons but also activates glial cells, both astrocytes and microglia. In previous studies with the gerbil model, a 5-min BCCA occlusion followed by reperfusion for 4 days led to prominent activation of astrocytes and microglial cells around the hippocampal CA1 region, where pyramidal neurons are extensively damaged (Wang et al., 2005a, 2009). With this murine model, we observed substantially greater activation of microglial cells at 3 days after a 30-min BCCA occlusion. The pattern of microglial cells activation reflects the areas where neurons are damaged. Alteration in microglial cell morphology toward the phagocytic form at this time after reperfusion is in agreement with the notion that microglia are actively responding to neuronal injury and cell death. The observation that mice consuming the Sutherlandia or elderberry diets showed significantly less microglial activation as compared with the ischemic brains of mice consuming control diet supports the capacity of these diets to mitigate neuron damage and microglial activation.

With the BCCA occlusion model, significant increase in astrocytes can be observed in the ischemic brain 3 days after I/R. Unlike the focal ischemia model where extensive astrogliosis is found in the penumbral area, astrocytes in the BCCA brain are more widespread in different brain regions. Furthermore, there is no significant difference in GFAP immunoreactivity comparing ischemic mice given the Sutherlandia or elderberry diets with control diets. These results further demonstrate effects of Sutherlandia and elderberry to protect ischemic damage through inhibiting neuron cell death and microglial cell activation. Although these results suggest an intimate relationship between neuronal damage and activation of microglial cells, more studies are needed to better understand mode of communication between these two cell types.

Increase in oxidative stress is an important factor in reperfusion injury; and several studies have implicated the involvement of NADPH oxidase as an important source of ROS (Chen et al., 2009; Yoshioka et al., 2011a). Although mechanisms for ROS produced by NADPH oxidase in neurons and glial cells are not well understood, our and other’s studies (Brennan et al., 2009) demonstrated rapid production of ROS in primary neurons (minutes) on stimulation by the ionotropic glutamate receptor agonist (*N*-methyl-d-aspartic acid, NMDA; Shelat et al., 2008); however, production of ROS in microglial cells follows a delayed process in hours (Chuang et al., 2013). When botanicals such as honokiol and Sutherlandia extract were used to test antioxidative effect on neurons (stimulated with NMDA) and microglial cells (stimulated with LPS), neurons were more sensitive to the antioxidative action than microglial cells (Chuang et al., 2013; Jiang et al., 2014). The role of NADPH oxidase in ROS production in neurons was demonstrated by using neurons from p47phox-deficient mice, which showed diminished response to ROS production in response to excitotoxic agents (Brennan et al., 2009). In the mouse model of global cerebral ischemia, an increase in p47phox immunoreactivity was observed in mouse striatum 3 days after I/R (Yoshioka et al., 2011a). In the present study, we further localize the I/R-induced increase in p47phox immunoreactivity to microglial cells. Again, the significantly lower expression of p47phox immunoreactivity in mice given the Sutherlandia and elderberry diets as compared with that given the control diet is in agreement with the observation of decreased microglia activation.

It has been reported that I/R stimulates activation of MAPK pathways, in particular, the p38 MAPK and the Ras/MEK signaling (Nito et al., 2008), which are attributed to activation of the aquaporin-4 channel responsible for astrocyte swelling (Nito et al., 2012). Other studies also demonstrated upregulation of the MAPK/ERK1/2 pathway in brain after stroke (Sawe et al., 2008; Yenari et al., 2010). In fact, ERK1/2 is regarded as the most important member of the MAPK family capable of mediating a range of cellular responses, including motility, inflammation, and cell survival as well as cell death (Sawe et al., 2008). In our recent study with microglial cells, IFNγ not only stimulates the canonical Janus kinase-Signal Transducer and Activator of Transcription (JAK-STAT) pathway but also the MAPK/ERK1/2 pathway, and in turn, phospho-ERK1/2 is linked to activation of a number of cytoplasmic proteins including p47phox of NADPH oxidase for ROS and inducible nitric oxide synthase (iNOS) for nitric oxide (NO) production (Chuang et al., 2013). Subsequently, inhibition of phospho-ERK1/2 by U0126 abrogated IFNγ-induced NO and ROS production pin a dose-dependent manner (Chuang et al., 2013). Our recent study also demonstrated the capacity of Sutherlandia extracts to inhibit IFNγ-induced phospho-ERK1/2 and subsequently mitigate ROS and NO production in microglial cells (Jiang et al., 2014). Botanical polyphenols, for example, the active ingredient of green tea, epigallocatechin-3-gallate, also attenuates NO production through downregulation of ERK1/2-associated proteins including ALDH2, ANXA1, and LGALS1 in LPS-stimulated BV-2 microglial cells (Qu et al., 2014). Other studies also demonstrate a critical role of the MAPK/ERK pathway in neuron excitation (Simon et al., 1984) and MEK/ERK inhibitors mitigating brain damage in the stroke model (Wang et al., 2003, 2004; Gladbach et al., 2013). Results of this study further support the important role of phospho-ERK1/2 expression in microglial cells after I/R, and suppression in mice fed diets supplemented with Sutherlandia and elderberry.

Because Sutherlandia is widely used in southern Africa for symptoms of HIV/AIDS and elderberry dietary supplements are among top selling products in Europe and North America, these studies provide new insights into use of these herbs as neuroprotective agents. In summary, we have demonstrated significant protective effects of dietary elderberry and Sutherlandia against global cerebral ischemia-induced functional motor deficits and neuropathological changes, including neuronal cell death and microglial activation. Results further support the hypotheses that these botanicals exert beneficial effects against ischemic damage through suppression of oxidative and proinflammatory pathways in neurons and microglial cells. This study provides strong rationale to further investigate the active components and mechanisms of action and to determine whether their consumption ameliorates ischemic damage as well as neurodegenerative diseases.

## Summary

This study demonstrates that Sutherlandia and American elderberry botanicals ameliorate ischemia/reperfusion (stroke)-induced behavioral dysfunction, neuronal damage, and oxidative stress and inflammatory responses in microglial cells.
